# Medulloblastoma in an Adult Female Patient: A Rare Presentation

**DOI:** 10.7759/cureus.16713

**Published:** 2021-07-29

**Authors:** Shobha Mandal, Bishnu Singh, Sumit Gami, Sunil Shah, Joyson Poulose

**Affiliations:** 1 Internal Medicine, Guthrie Robert Packer Hospital, Sayre, USA; 2 General Medicine, Himal Hospital Private Limited, Kathmandu, NPL; 3 Medicine, Universal College of Medical Sciences, Bhairahawa, NPL; 4 Internal Medicine, Patan Academy of Health Sciences, Patan, NPL; 5 Medicine, Ministry of Health, Malé, MDV; 6 Medicine, California Institute of Behavioral Neurosciences & Psychology, Fairfield, USA; 7 Hematology and Oncology, Guthrie Robert Packer Hospital, Sayre, USA

**Keywords:** adult medulloblastoma, rare cancer, central nervous system, cerebellum, children

## Abstract

Medulloblastoma (MB) is an aggressive malignant tumor of the posterior fossa of the CNS that mainly affects children younger than 15 years of age. It is uncommon in the adult population compared to children. Any adult patient presenting with cerebellar mass must be evaluated with brain tissue biopsy to rule out MB. Our patient is a 27-year-old female who presented with sudden onset of frontal headache and was diagnosed with MB.

## Introduction

Medulloblastoma (MB) is an aggressive neoplasm of embryonal origin. It is most commonly located in the vermis of the cerebellum and commonly affects children [1]. It accounts for 30% of pediatric CNS neoplasms, whereas it is rare in adults with an annual incidence rate of 0.05/100,000 per year. Nearly 75% of MB occurs in children under the age of 10 [2]. Patients have a variable initial presentation including nocturnal or morning headaches, nausea, vomiting, and altered mental status. On head imaging, findings are variable, hence, the histopathological examination must be performed to confirm the diagnosis.

## Case presentation

**Figure 1:**
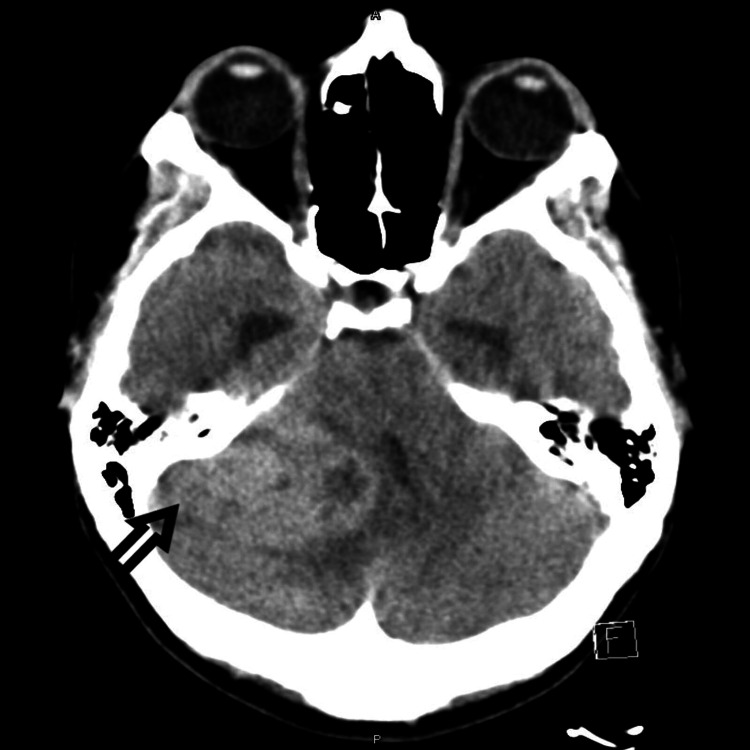
CT scan of the head without intravenous contrast showing a large right cerebellar mass with midline shift in the posterior fossa (shown by arrow).

**Figure 2:**
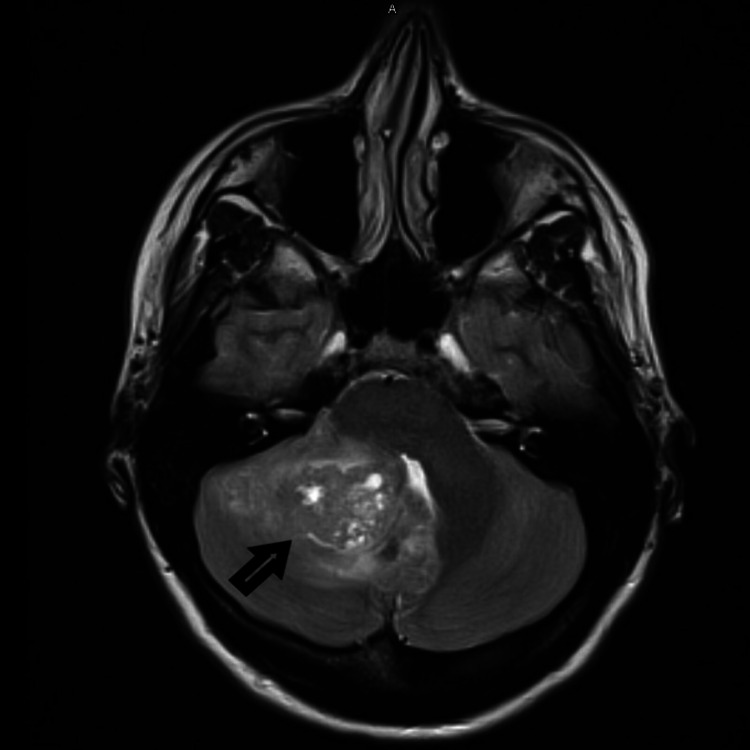
MRI of the brain showing right medial cerebellum mass.

A 27-year-old female with a past medical history of migraine headache presented to the ED with a complaint of sudden onset frontal headache, different from her usual migraine headache. Headache was frontal, worse in the night and morning, not relieved with over-the-counter acetaminophen. The physical exam was normal, and vitals were stable. CT scan of the head without intravenous contrast showed a large right cerebellar mass measuring 1.4 x 2.2 x 1.5 cm with a midline shift in the posterior fossa (Figure 1). MRI of the brain with and without contrast showed enhancing mass in the right cerebellum with cysts and necrosis measuring 2.4 x 2.7 x 2.1 cm confirmed the findings (Figure 2).

**Figure 3:**
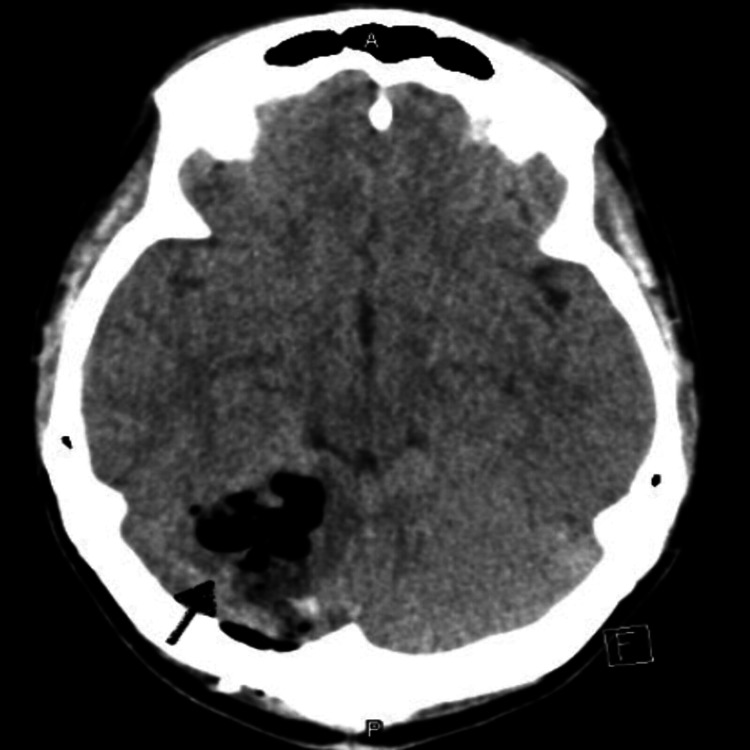
CT without contrast of head after the second resection of the tumor.

**Figure 4:**
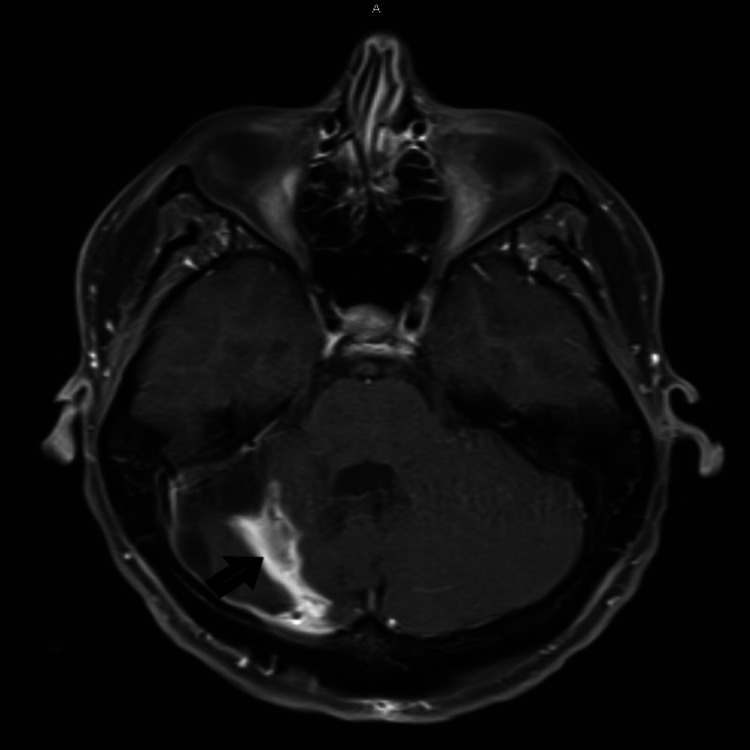
MRI of the brain showing finding suggestive of postoperative changes to the right cerebellum.

**Figure 5:**
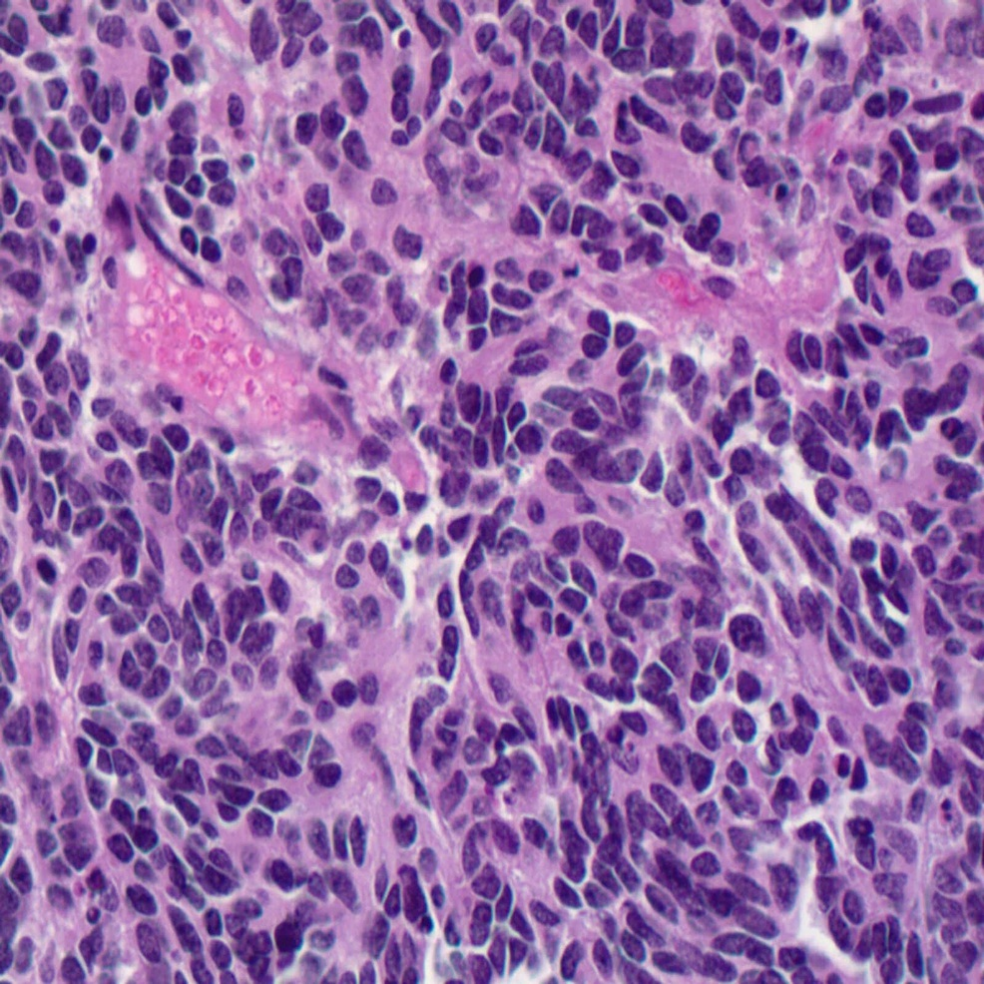
Immunohistochemistry showing sheet-like proliferation of small blue cells with high nuclear to cytoplasmic ratio, finely stippled chromatin pattern, and inconspicuous nucleoli associated with Homer Wright rosettes (H&E X400).

**Figure 6:**
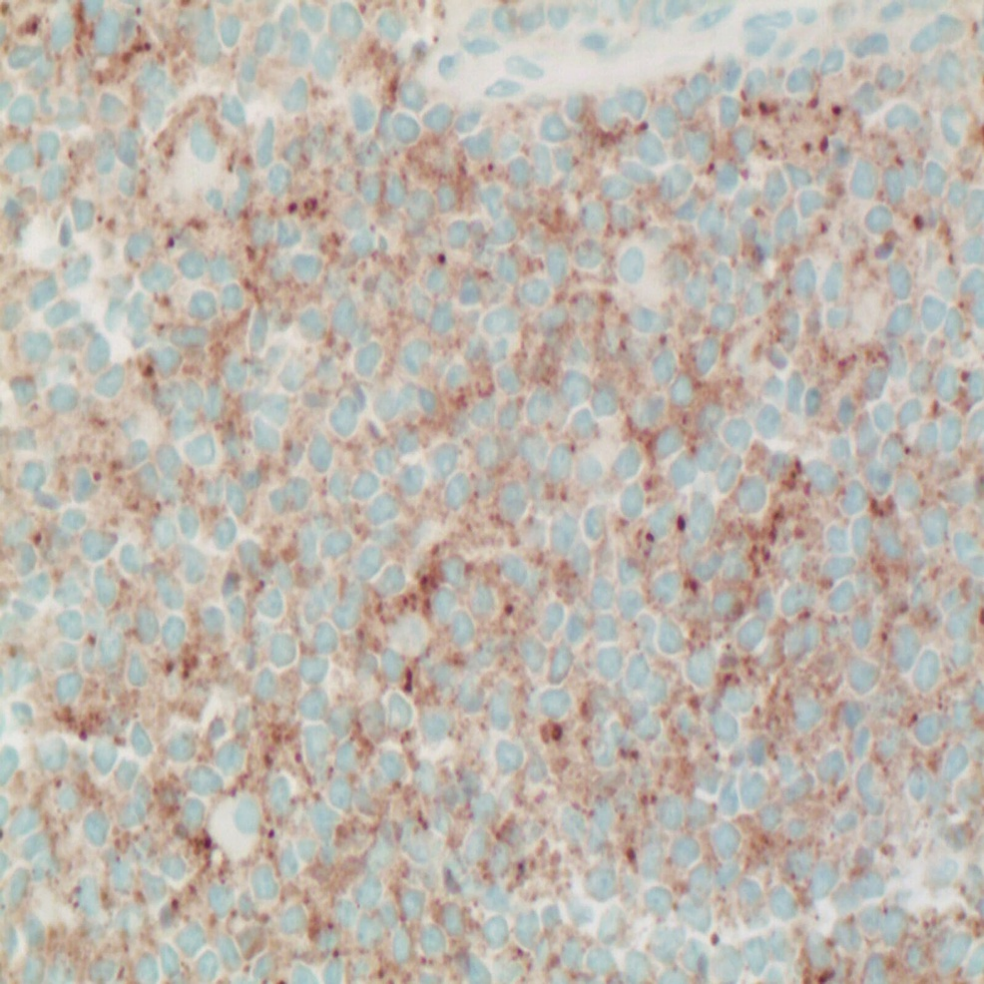
Immunohistochemistry showing tumor cells positive for synaptophysin (X400).

**Figure 7:**
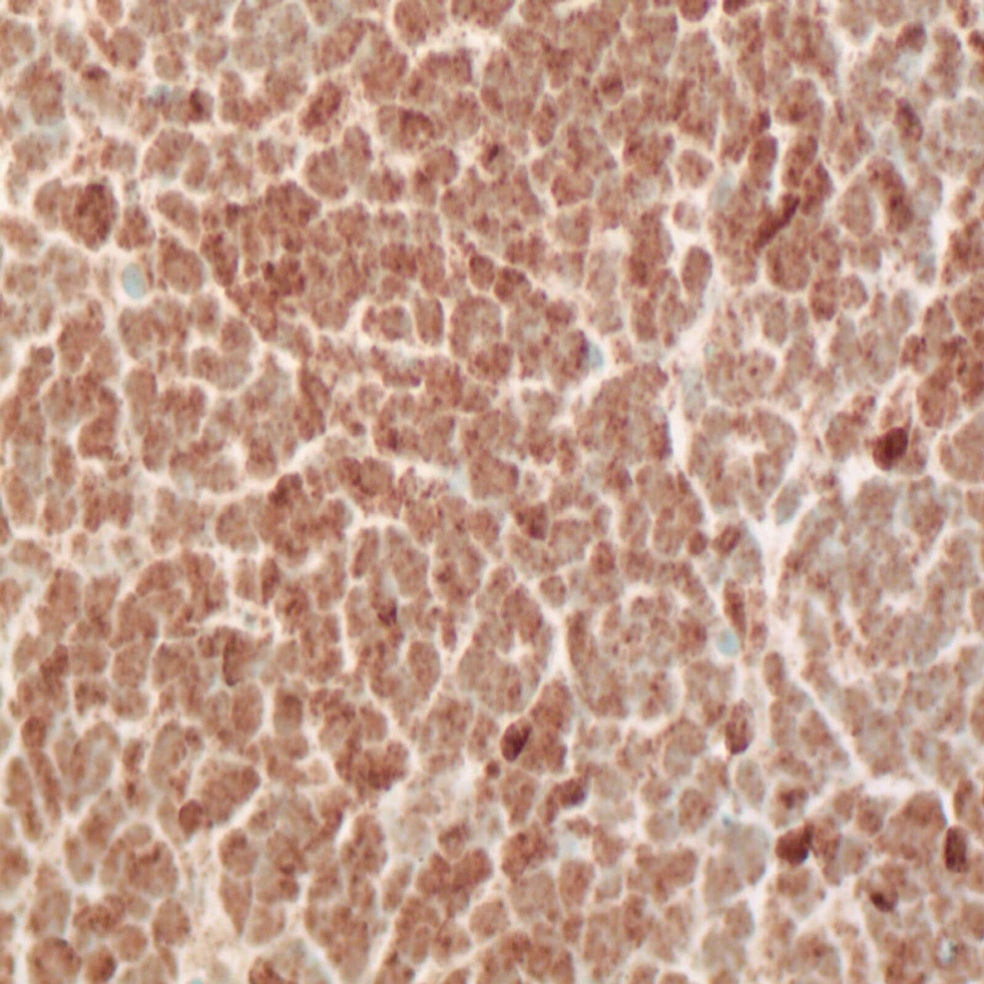
Immunohistochemistry showing tumor cells positive for YAP1 (X400). YAP1: Yes-associated protein 1.

The neurosurgery team was on board and the patient was planned for initial partial resection of the tumor and underwent right suboccipital craniotomy with partial resection of the tumor. She was followed up in two weeks and underwent a repeat craniotomy and resection of the remaining tumor without any complication. Postoperative CT scan and MRI showed no obvious evidence of residual disease, consistent with a gross total resection (Figures 3-4). Histopathology of the tumor was consistent with classic sonic hedgehog (SHH)-activated medulloblastoma (composed of blue cells with focal formation of Homer Wright rosettes) (Figure 5). The additional molecule was positive for synaptophysin (Figure 6). The tumor cells showed expression of yes-associated protein 1 (YAP1) (Figure 7).

Postresection, the patient felt well except for some residual right-hand weakness. The cerebrospinal fluid (CSF) analysis cytology was negative for malignant cells. According to National Comprehensive Cancer Network (NCCN) guidelines, treatment protocol included maximal safe resection, followed by adjuvant therapy involving chemotherapy and radiation. The plan was to start the patient on adjuvant craniospinal radiotherapy with concurrent vincristine for a period of eight weeks, followed by maintenance multiagent chemotherapy including cisplatin, lomustine, and vincristine for eight cycles.

## Discussion

MB is most commonly located in the vermis of the cerebellum in children but can involve the lateral hemispheres of the cerebellum in adults. It occurs more frequently in men than women [3]. Metastasis is less common in adults compared to children [4]. It is found to spread through CSF in the CNS. It involves the CNS and extraneural metastases are rare. If it involves the extraneural sites, bone is the most common site of metastasis whereas sometimes it can also involve the lung or liver. It is found to be associated with multiple rare genetic disorders including Gorlin syndrome, Li-Fraumeni syndrome, APC-associated polyposis, and Fanconi anemia [5].

Patients with MB classically present with clinical signs and symptoms of increased intracranial pressure like night and early morning headache, nausea, vomiting, confusion, and blurring of vision. Tumors located in the midline can manifest as gait ataxia or truncal instability, whereas those located in the lateral cerebellar hemisphere cause limb clumsiness or incoordination [6]. It can also infiltrate the cranial nerves and involvement of cranial nerve IV or VI leads to Nystagmus and diplopia. On physical exams, patients may show cerebellar signs like dysmetria on finger-to-nose testing, intention tremor, and difficulty with heel-to-shine testing. On funduscopic examination, papilledema can be seen [6]. 

Apart from physical exams, CT and MRI of the brain are needed to support the diagnosis. MRI shows iso- or hypointense on T1-weighted images and hyper to hypointense on T2-weighted images [7]. Hydrocephalus may be present secondary to obstruction at the level of the fourth ventricle. Adult MBs are more likely to demonstrate inhomogeneous contrast enhancement, a hyperintense signal on T1, and a hypointense signal on T2 sequences and are less likely to demonstrate contrast enhancement when compared with the pediatric MB [8]. CT scan is not the best imaging as it can miss the diagnosis. Nearly one-third of MBs metastasize throughout the CNS following CSF pathways, hence CSF analysis may show elevated protein, mild pleocytosis, and can have positive cytology but negative cytology does not exclude the diagnosis. Confirmatory diagnosis can be done with histopathology confirmation.

MB is classified into several variants by the WHO classification of brain tumors which divides the tumor based upon histopathologic criteria. Different variants of MB are classic, desmoplastic/nodular, desmoplastic with extensive nodularity, large cell, or anaplastic. Among these variants, classic MB is the most common variant among both children and adults (70-80%) and extensive nodularity is the least common one (3%) [9]. Histologically, it has densely packed small round undifferentiated cells with mild-to-moderate nuclear pleomorphism and a high mitotic count [10]. MB can be further classified into four distinct molecular subgroups based on transcriptome profiling and are designated as WNT-activated, SHH-activated, group 3, and group 4. SHH being the most common in adults. Histologically, adult patients and infants are more commonly affected by the desmoplastic/nodular variant, whereas children are affected by the classic variant [11]. Patients are categorized into average vs high risk depending on the residual tumor on MRI, histology variants, CSF involvement. Adults with residual tumor <1.5 cm2, negative spine MRI and CSF cytology, and classic or desmoplastic histology are considered average risk whereas those with bulky residual disease, evidence of leptomeningeal dissemination or distant metastasis, large cell or anaplastic histology, and molecular marker positive for MYC amplification, SHH tumor are considered high risk [12,13].

As MB in adults is rare, there is no clear guideline for treatment in adults. Treatment is based on guidelines in children itself [14]. The aim of treatment is to relieve the increased intracranial pressure and tumor-directed specific therapy. The most major step in the tumor-specific treatment is maximal safe resection followed by craniospinal irradiation. Postoperative brain MRI is recommended within 48 hours of surgery to assess for the residual tumor. Radiotherapy assists in controlling residual posterior fossa tumors and treating the metastatic tumor of the craniospinal axis. In patients with metastatic disease, chemotherapy after the completion of radiation therapy can be considered [15]. The most commonly used regimen is the packer regimen, which includes platinum-based chemotherapy (combination of vincristine, cisplatin, cyclophosphamide, or lomustine) [16]. As we do not have clear trials on adult treatment with chemotherapy, we do not know whether it is as beneficial in adults as in children, hence chemotherapy is often reserved for high-risk adult patients [11,12]. Recently, immunotherapy has gained popularity in the treatment of MB in adults. It aims to block immune checkpoint inhibitors such as programmed death-1 (PD-1) and cytotoxic T-lymphocyte-associated protein 4 (CTLA-4), and decrease the immune response [17]. 

In a high-risk patient, with positive CSF cytology, combination treatment with standard-dose craniospinal radiotherapy with posterior fossa boost followed by multiagent maintenance chemotherapy is considered effective [18]. Those patients who have the recurrent disease may benefit from resection of localized brain tumor recurrence followed by additional chemotherapy or focal radiotherapy. Recently smoothened (SMO) inhibitors like vismodegib are new therapeutics for patients with SHH-MB. It was well tolerated in patients with MB in phase II trials and the precursor phase I trial [11] but its toxicity is not well understood hence needs monitoring [19].

With the evolving treatment modalities of MB, the prognosis is variable in both adults and children. In patients who received postoperative cranioradiation therapy, the prognosis was favorable for adults compared to children. Adult patients who have group 4 tumors are found to have poor prognoses compared to children. A desmoplastic variant has a better prognosis compared to the classic variant. Those patients who have positive CSF cytology are found to have an increased rate of relapse and poor outcomes. Any patient with spinal seeding at presentation also has a poor prognosis [10].

## Conclusions

MB is the most common brain tumor in children but is rare in adults. Every patient with posterior fossa mass must undergo a biopsy of the mass with histopathological and immunohistochemical examination to confirm the diagnosis, as radiographic imaging alone could be inadequate.
